# Prevalence, phenomenology and personality characteristics of premenstrual dysphoric disorder among female students at Zagazig University, Egypt

**DOI:** 10.4102/phcfm.v13i1.2924

**Published:** 2021-08-30

**Authors:** Seham M. Eldeeb, Afaf M. Eladl, Amany Elshabrawy, Amira M. Youssef, Mona H. Ibrahim

**Affiliations:** 1 Department of Community, Environmental and Occupational Medicine, Faculty of Medicine, Zagazig University, Zagazig, Egypt; 2 Department of Psychiatry, Faculty of Medicine, Zagazig University, Zagazig, Egypt

**Keywords:** premenstrual dysphoric disorder, premenstrual phenomena, university students, prevalence, personality characteristics

## Abstract

**Background:**

Premenstrual dysphoric disorder (PMDD) is a female psychiatric disorder affecting the behaviour, cognitive abilities, mental health status and academic performance of female students. It includes: mood symptoms, behaviour symptoms and physical symptoms.

**Aim:**

To assess phenomenology, measure the prevalence of PMDD among university students and assess the relationship between PMDD and socio-demographic and personality characteristics.

**Setting:**

This study was conducted at Zagazig University, Sharqia Governorate, Egypt.

**Methods:**

A cross-sectional study was conducted from September 2020 to December 2020. It included 755 university students. They filled several questionnaires covering Diagnostic and Statistical Manual of Disorders (DSM-5) criteria to diagnose PMDD, socio-demographic, menstrual factors, physical activity and personality traits.

**Results:**

Premenstrual dysphoric disorder was found in 159 out of 755 students (21.1%). Overall, the most frequently reported premenstrual symptoms were overeating/food cravings (84.2%), fatigue/lack of energy (83.6%), depressed mood/hopelessness (82.0%) and hypersomnia (78.9%). Binary logistic regression model revealed that significantly related PMDD risk factors include: being a medical student, having a duration of menstrual bleeding ≥ 7 days, the average length of one cycle ˂ 28 days, high menstrual blood loss, presence of dysmenorrhea and positive family history of premenstrual syndrome (sister/mother). Regarding personality traits, low extroversion and agreeableness, and high neuroticism were also significant PMDD risk factors.

**Conclusion:**

Prevalence of PMDD was high among university students, especially medical students, and it can have a detrimental effect on both academic life and educational accomplishments, quality of life and daily living activities.

## Introduction

Premenstrual dysphoric disorder (PMDD) is also referred to as late luteal phase dysphoric disorder. Mood symptoms, behaviour symptoms and physical symptoms are involved in the syndrome. This pattern of symptoms is seen during the menstrual cycle at a particular moment, and for a while, between menstrual cycles, the symptoms resolve. In female students in higher education institutions, it is one of the most common problems that has negatively affected academic performance and professional and interpersonal relationships.^1^

Mental health problems that result from or are associated with menstruation are critical gender-related mental health disorders in women. Especially in the luteal phase, menstrual changes are linked to various cognitive, behavioural and psychological symptoms called premenstrual symptoms.^2^

While premenstrual syndrome (PMS) generally refers to any somatic or psychological symptoms that affect functioning, PMDD is a newly recognised psychiatric disorder that affects 1%–8% of women, often conceptually placed at the extreme end of premenstrual symptom severity.^3^ Premenstrual dysphoric disorder has strict diagnostic criteria implemented in the Diagnostic and Statistical Manual (DSM-5) of the American Psychiatric Association in 2013.^4^

While the etiology remains unknown, some studies have suggested that premenstrual problems are bio-psycho-social.^5^ There is general agreement that premenstrual symptoms may occur because of increased sensitivity of the central nervous system to menstrual cycle related hormonal fluctuations.^6^

Concerning psychological factors, some studies have suggested that PMDD is associated with personality characteristics. Elevations of specific personality dimensions, such as harm-avoidance and novelty-seeking, neuroticism and perfectionism, have been reported to harm existing severe premenstrual changes.^7^

University students are at an age when there is a lack of psychological and social capacity to manage their daily stressors. They strive for higher academic achievement to secure better jobs and satisfy their own needs for realisation. At the same time, they lack resources and are burdened by psychological and social demands for example self-esteem and social support (friends and family).^8^ However, university students do not seek psychological or social assistance for fear of stigma. Perception of consequences of a mental health problem affects the confession of its presence. The consequences include general beliefs about the impact of the illness on the patient’s personal life, family, social relationships and finances, and how disabling the disease is likely to be.^9^

Therefore, defining and resolving their concerns is very relevant. Around half of Egyptian university students are female, and their educational performance and other aspects of their lives may be affected by premenstrual issues. The prevalence of premenstrual syndrome in Egypt ranges from 80.0% to 92.3% of girls aged 12–25 years.^10^ Despite the high prevalence of PMS and PMDD in Egypt, examining the relationship between personality characteristics and PMS and PMDD has received little attention. Therefore, this study aimed to study phenomenology, measure the prevalence of PMDD among university students and assess the relationship between PMDD and socio-demographic and personality characteristics.

## Methods

### Study design and setting

This research utilised a cross-sectional design and was conducted at Zagazig University, a public university located in Zagazig, Egypt. The study includes 25 faculties and institutes (13 practical and 12 theoretical). According to Webometrics, Universities rank (2020), Zagazig University is at the 24th position in Africa and at the 7th position at the national level.

### Study population

Female undergraduate students at Zagazig University, in all grades, during the study period, who agreed to participate in the study were included. However, pregnant, lactating females have irregular periods (duration of the cycle is either less than 24 days or more than 35 days), and those taking hormonal contraceptives, substance abuse, having a history of any psychiatric disorders other than PMDD, somatic diseases, gynaecological or hormonal disorders were excluded.

### Sample size and sampling

Provided that the total number of female students in Zagazig university during the academic year 2019–2020 was 82 538, the prevalence of PMDD is 7.7%,^11^ confidence limit is 3.8% and design effect is 4. At the confidence level, 95% of the total sample size was 755 students. The values were calculated using Epi Info 7 version 7.2.0.1.^12^

Zagazig University has 25 faculties and institutes (13 practical and 12 theoretical). First, by random selection, we choose two faculties. Then, a cluster sample of a section was randomly selected from each grade. Finally, proportional allocation was considered when determining the number of students from each faculty included in our study.

### Tools and data collection

The participants completed questionnaires directly on Google Forms; each questionnaire was sent to the final database and downloaded as a Microsoft Excel sheet. The Google Form was distributed to the selected sections through the official e-learning panels. The participants’ answers were anonymous and confidential, according to Google’s privacy policy.^13^ The participants were able to withdraw their participation in the survey at any stage before the submission; non-completed responses were not saved. The survey includes an introductory page describing the background, aims and information on the survey’s ethics.

Five tools were used to collect the necessary data about the study subjects as the following:

**First tool:** Socio-economic characteristics of study participants:

The socio-economic status was measured by scale for measuring family socio-economic status (SES) for health research in Egypt.^14^ This scale includes seven domains: education of both father and mother, occupation of both father and mother, family domain, family possessions domain, home sanitation domain, economic domain and healthcare domain. The total score was 84. The socio-economic status was classified as low, if total score was less than 43; middle, if total score was between 43 and 62 and high, if total score was above 62.

**Second tool:** Menstrual blood loss (MBL):

Menstrual blood loss (MBL) is a previously validated, structured scale^15^ estimating MBL. The MBL-score is based on three items of the woman’s menstrual period obtained from a questionnaire filled out during menstruation and not from memory. Menstrual blood loss is estimated by counting the number of pads then multiplying them with absorbance numbers according to the manufacturer’s reported absorbance level. The absorbance numbers for pads were: mini 1, normal 1.5, super 2, night/superplus 3. Tampons are not popular among girls in Egypt, and none of the study participants reported using tampons. Menstrual blood loss-score was calculated as follows:

MBL-score=(Number of heavy days÷Number of days of menstruation)×MBL [Eqn 1]

**Third tool:** Sedentary Behaviour Questionnaire (SBQ):

Sedentary Behaviour Questionnaire (SBQ) is a previously validated, structured scale^16^ estimating the amount of time spent performing nine common sedentary behaviour forms (watching television, playing computer or video games, sitting while listening to music, sitting and talking on the phone, doing paperwork or office work, sitting and reading, playing a musical instrument, doing arts and crafts and sitting and driving/riding in a car, bus or train). The nine items are completed separately for weekdays and weekends. Participants respond to the question, ‘On a typical weekday (or weekend day), how much time do you spend (from when you wake up until you go to bed) doing the following?’ The available response options are: none, ≤ 15 minutes, 30 minutes, 1 hour, 2 hours, 3 hours, 4 hours, 5 hours or ≥ 6 hours. Average sedentary hours across all days were calculated using a weighted average:

(weekday hours×5)+(weekend hours×2)÷7. [Eqn 2]

**Fourth tool:** Premenstrual Symptoms Screening Tool (PSST):

This is an instrument designed by Özdel et al. (2015)^17^ to identify the women who suffer from PMDD based on modified DSM-5 criteria. It consists of two sections:

- The first section is a checklist that consists of 14 items (from 1 to 14) that inquire about the experience of the premenstrual symptoms. Students were asked, ‘Do you experience some or any of the following premenstrual symptoms which start before your period and stop within a few days of bleeding?’ The symptoms listed are anger/irritability, anxiety/tension, tearful/increased sensitivity to rejection, depressed mood/hopelessness, decreased interest in studying activities, decreased interest in home activities, decreased interest in social activities, difficulty concentrating, fatigue/lack of energy, overeating/food cravings, insomnia, hypersomnia, feeling overwhelmed or out of control and physical symptoms.
- The second section consists of five items (A, B, C, D and E). The students were asked if these symptoms interfere with study efficiency or productivity, relationships with co-workers, relationships with their families, social life activities and home responsibilities. The two sections are rated on a five-point scale (from 1 = not at all to 4 = severe). For a diagnosis of PMDD, the following must be present:
  ■ At least one of the items of 1, 2, 3 and 4 is severe.
  ■ Besides, at least four of 1 to14 are moderate to severe
  ■ At least one of A, B, C, D, E is severe

**Fifth tool:** Big five personality traits inventory (BFI):

This scale was planned by John & Srivastava^18^ and measures big five personality traits and comprises 44 items that evaluate five basic personality traits: extroversion, conscientiousness, agreeableness, neuroticism and openness to experience. Extroversion is assessed using eight adjectives, including: ‘timid, withdrawn, shy, talkative, lethargic, enterprising, cold and passive’; conscientiousness is assessed using eight adjectives, including: ‘self-disciplined, tidy, hard-working, prudent, fussy, determined, irresponsible and lazy’; agreeableness is assessed using eight adjectives including: ‘sincere, compassionate, genial, well-intentioned, philanthropic, tolerant, sharer and sensitive’; neuroticism is assessed using nine adjectives including: ‘nervous, aggressive, angry, temperamental, impatient, capricious, impetuous, touchy and worried’; openness to experience is assessed using six adjectives including: ‘self-confident, self-assured, brave, creative, easy going and capable’. The BFI is rated on a 5-points Likert scale from 1 (disagree a lot) to 5 (agree a lot).

### Content validity and reliability

The tools were tested for content validity by the panel comprising five experts of the Psychiatry Department. These experts assessed the tools for clarity, relevance, comprehensiveness, applicability and understanding. All tools were translated into Arabic language using the translate–back-translate technique to ensure their original validity. A pilot study was conducted 1 month before data collection to detect any difficulties and test the questionnaire’s reliability after translation. The sample included in the pilot study was excluded from the main sample because of the changes in the final version of the questionnaire.

### Statistical analysis

Statistical analysis was conducted using Statistical Package for the Social Sciences (SPSS) software version 27 (IBM, 2020).^19^ Kolmogorov–Smirnov and Levene tests were used to determine the distribution characteristics of variables and variance homogeneity. Descriptive statistics were generated for all variables. Comparison between students with PMDD and without PMDD was done using the appropriate tests of significance. Student’s *t* test was used to analyse continuous variables. Pearson’s chi squared test, and chi square for linear trend were used to analyse qualitative variables as appropriate. All the factors that were significantly associated with PMDD were run in a binary logistic regression model to determine the independent predictors of PMDD. The level of statistical significance was set at *p* < 0.05.

## Results

A total of 755 participants were included in the study. Socio-demographic, menstrual and personality characteristics of the study participants are presented in Table 1.

**TABLE 1:** Socio-demographic, menstrual and personality characteristics of the study participants.

Variables	Study participants (*N* = 755)				
	Median	Range	Mean ± s.d.	*n*	%
**Age (years)**	-	-	21.2 ± 3.7	-	-
Faculty	-	-	-	-	-
Commerce	-	-	-	565	74.8
Medicine	-	-		190	25.2
**Residence**					
Rural	-	-	-	490	65.0
Urban	-	-	-	265	35.0
**Marital status**					
Single	-	-	-	647	85.7
Married	-	-	-	108	14.3
**Socio-economic class**					
Low	-	-	-	116	15.4
Middle	-	-	-	407	53.9
High	-	-	-	232	30.7
**Body mass index**					
˂ 30 kg/m^2^ (Non-obese)	-	-	-	574	76.0
≥ 30 kg/m^2^ (Obese)	-	-	-	181	24.0
**Age of menarche**					
˂ 12 years old	-	-	-	102	13.5
12–14 years old	-	-	-	463	61.3
˃ 14 years old	-	-	-	190	25.2
**Duration of menstrual bleeding**					
< 7 days	-	-	-	486	64.4
≥ 7 days	-	-	-	269	35.6
**The average length of one cycle**					
˂ 28 days	-	-	-	249	33.0
≥ 28 days	-	-	-	422	55.9
Irregular	-	-	-	84	11.1
**Menstrual bleed loss (MBL)**					
Low (MBL-score ≤ 4)	-	-	-	364	48.2
High (MBL-score ˃ 4)	-	-	-	391	51.8
**Dysmenorrhea**					
Yes	-	-	-	671	88.9
No	-	-	-	84	11.1
**Family history of premenstrual syndrome (sister/mother)**					
Positive	-	-	-	625	82.8
Negative	-	-	-	130	17.2
Sedentary behaviour hours per day	6.7	4.4– 11.8		-	-
**BFI personality traits**					
Extroversion	-	-	25.6 ± 4.6	-	-
Agreeableness	-	-	35.4 ± 2.7	-	-
Conscientiousness	-	-	30.8 ± 3.5	-	-
Neuroticism	-	-	23.1 ± 4.5	-	-
Openness	-	-	32.2 ± 2.8	-	-

s.d., standard deviation; BFI, big five personality traits inventory; s.d., standard deviation.

**FIGURE 1:**
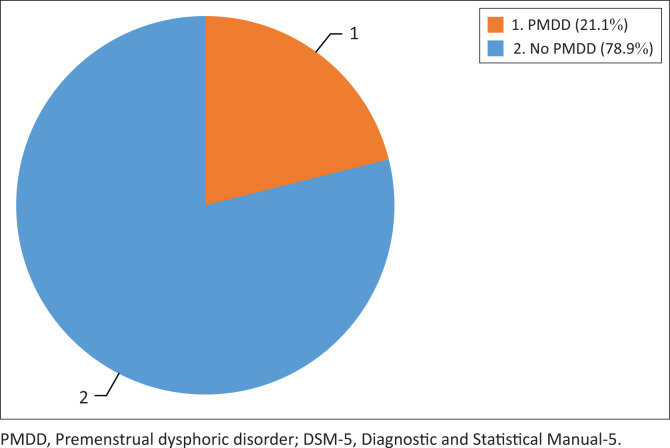
Prevalence of premenstrual dysphoric disorder (according to DSM-5 criteria) among the study participants.

Premenstrual dysphoric disorder was found in 159 out of 755 students (21.1%) (Figure 1). Generally, mild premenstrual symptoms were the most frequently reported, followed by moderate symptoms. Severe symptoms were the least frequently reported. Almost two-thirds of the study participants had at least one premenstrual symptom with varying degree of severity (Table 2).

**TABLE 2:** Frequency distribution of the study participants according to the symptomatology of premenstrual dysphoric disorder.

Severity	Not at all		Mild		Moderate		Severe	
	*n*	*%*	*n*	*%*	*n*	*%*	*n*	*%*
**Symptoms^†^**								
1. Anger/irritability	172	22.8	242	32.0	193	25.6	148	19.6
2. Anxiety/tension	184	23.8	285	36.9	187	24.2	117	15.1
3. Tearful/increased sensitivity to rejection	244	32.3	307	40.7	149	19.7	55	7.3
4. Depressed mood/hopelessness	136	18.0	261	34.6	199	26.4	159	21.0
5. Decreased interest in studying activities	238	31.7	241	32.1	170	22.7	101	13.5
6. Decreased interest in home activities	163	21.6	314	41.6	196	26.0	82	10.8
7. Decreased interest in social activities	209	27.7	226	29.9	183	24.2	137	18.2
8. Difficulty concentrating	192	25.4	287	38.0	165	21.9	111	14.7
9. Fatigue/lack of energy	124	16.4	231	30.6	262	34.7	138	18.3
10. Overeating/food cravings	119	15.8	270	35.7	238	31.5	128	17.0
11. Insomnia	276	36.6	306	40.5	158	20.9	15	2.0
12. Hypersomnia	159	21.1	242	32.0	181	24.0	173	22.9
13. Feeling overwhelmed or out of control	241	31.9	296	39.2	179	23.7	39	5.2
14. Physical symptoms: breast tenderness, headache, joint/muscle pain, bloating, weight gain	168	22.3	271	35.9	168	22.2	148	19.6
**The symptoms listed above interfered with:**								
A. Studying efficiency or productivity	162	21.5	274	36.3	198	26.2	121	16.0
B. Relationships with colleagues	201	26.6	236	31.3	189	25.0	129	17.1
C. Relationships with family	147	19.5	264	35.0	203	26.9	141	18.7
D. Social life activities	172	22.8	249	33.0	205	27.1	129	17.1
E. Home responsibilities	237	31.4	311	41.2	184	24.4	23	3.0

† , Starts before period and stops within a few days of bleeding.

Overall, the most frequently reported premenstrual symptoms were overeating/food cravings (84.2%), fatigue/lack of energy (83.6%), depressed mood/hopelessness (82.0%) and hypersomnia (78.9%).

The most frequent severe symptoms were hypersomnia (22.9%), followed by depressed mood/hopelessness (21.0%), physical symptoms, for example breast tenderness, headache, joint/muscle pain, bloating, weight gain (19.6%) and fatigue/lack of energy (18.3%).

The most frequent moderate symptoms were fatigue/lack of energy (34.7%), overeating/food cravings (31.5%), depressed mood/hopelessness (26.4%) and decreased interest in home activities (26.0%).

The most frequent mild symptoms were decreased interest in home activities (41.6%), tearful/increased sensitivity to rejection (40.7%), insomnia (40.5%) and feeling overwhelmed or out of control (39.2%).

The least frequent symptoms (frequently reported as ‘not at all’) are insomnia, tearful/increased sensitivity to rejection, feeling overwhelmed or out of control and decreased interest in studying activities.

The premenstrual symptoms interfered mainly with family relationships followed by relationships with colleagues, social life activities, then studying efficiency or productivity (severe in 18.7%, 17.1%, 17.1% & 16.0%, respectively). Home responsibilities were the least to be affected (severe in 3.0% of the study participants).

There were statistically significant relations between PMDD and the study participants’ socio-demographic, menstrual and personality characteristics. Students with PMDD had higher age than students without PMDD (21.7 ± 3.4 vs 20.6 ± 3.8). Medical students had a higher prevalence of PMDD than commerce students (32.1% vs. 17.4%). Urban residents had a higher prevalence than rural residents (26.4% vs. 18.2%). Almost two-thirds of low socio-economic class students had PMDD. Obese students had a higher prevalence of PMDD compared to non-obese students (30.4% vs. 18.1%) (Table 3).

**TABLE 3:** Relation between premenstrual dysphoric disorder and both socio-demographic, menstrual and personality characteristics of the study participants.

Variables	PMDD (*n* = 159)					No PMDD (*n* = 596)					*p*
	Median	Range	Mean ± s.d.	*n*	%	Median	Range	Mean ± s.d.	*n*	%	
**Age (years)**	-	-	21.7 ± 3.4	-	-	-	-	20.6 ± 3.8	-	-	< 0.001*
**Faculty**	-	-	-	-	-	-	-	-	-	-	< 0.001*
Commerce (*n* = 565)	-	-	-	98	17.4	-	-	-	467	82.6	
Medicine (*n* = 190)	-	-	-	61	32.1	-	-	-	129	67.9	
**Residence**	-	-	-	-	-	-	-	-	-	-	0.008*
Rural (*n* = 490)	-	-	-	89	18.2	-	-	-	401	81.8	
Urban (*n* = 265)	-	-	-	70	26.4	-	-	-	195	73.6	
**Mar ital status**	-	-	-	-	-	-	-	-	-	-	0.7
Single (*n* = 647)	-	-	-	135	20.9	-	-	-	512	79.1	
Married (*n* = 108)	-	-	-	24	22.2	-	-	-	84	77.8	
**Socio-economic class**	-	-	-	-	-	-	-	-	-	-	< 0.001*
Low (*n* = 116)	-	-	-	76	65.5	-	-	-	40	34.5	
Middle (*n* = 407)	-	-	-	61	15.0	-	-	-	346	85.0	
High (*n* = 232)	-	-	-	22	9.5	-	-	-	210	90.5	
**Body mass index**	-	-	-	-	-	-	-	-	-	-	< 0.001*
˂ 30 Kg/m^2^ (*n* = 574)	-	-	-	104	18.1	-	-	-	470	81.9	
≥ 30 Kg/m^2^ (*n* = 181)	-	-	-	55	30.4	-	-	-	126	69.6	
**Age of menarche**	-	-	-	-	-	-	-	-	-	-	< 0.001*
˂ 12 years old (*n* = 102)	-	-	-	62	60.8	-	-	-	40	39.2	
12–14 years old (*n* = 463)	-	-	-	85	18.4	-	-	-	378	81.6	
˃ 14 years old (*n* = 190)	-	-	-	12	6.3	-	-	-	178	93.7	
**Duration of menstrual bleeding**	-	-	-	-	-	-	-	-	-	-	< 0.001*
< 7 days (*n* = 486)	-	-	-	75	15.4	-	-	-	411	84.6	
≥ 7 days (*n* = 269)	-	-	-	84	31.2	-	-	-	185	68.8	
**Average length of one cycle**	-	-	-	-	-	-	-	-	-	-	< 0.001*
˂ 28 days (*n* = 249)	-	-	-	98	39.4	-	-	-	151	60.6	
≥ 28 days (*n* = 422)	-	-	-	56	13.3	-	-	-	366	86.8	
Irregular (*n* = 84)	-	-	-	5	6.0	-	-	-	79	94.0	
**Menstrual bleed loss (MBL)**	-	-	-	-	-	-	-	-	-	-	< 0.001*
Low (*n* = 364)	-	-	-	43	11.8	-	-	-	321	88.2	
High (*n* = 391)	-	-	-	116	29.7	-	-	-	275	70.3	
**Dysmenorrhea**	-	-	-	-	-	-	-		-	-	< 0.001*
Yes (*n* = 671)	-	-	-	154	23.0	-	-	-	517	77.0	
No (*n* = 84)	-	-	-	3	6.0	-	-	-	79	94.0	
**Fam ily history of premenstrual syndrome (sister/mother)**	-	-	-	-	-	-	-	-	-	-	0.003*
Positive (*n* = 625)	-	-	-	119	19.0	-	-	-	506	81.0	
Negative (*n* = 130)	-	-	-	40	30.8	-	-	-	90	69.2	
**Sedentary behavior hours per day**	6.8	5.1–11.8	-	-	-	6.6	4.4–9.2	-	-	-	0.3
**BFI Personality**	-	-	-	-	-	-	-	-	-	-	-
Extroversion	-	-	24.5 ± 6.6	-	-	-	-	25.6 ± 4.5	-	-	0.04*
Agreeableness	-	-	36.4 ± 2.6	-	-	-	-	37.5 ± 1.7	-	-	< 0.001*
Conscientiousness	-	-	29.9 ± 7.6	-	-	-	-	30.4 ± 2.3	-	-	0.2
Neuroticism	-	-	24.4 ± 5.2	-	-	-	-	21.1 ± 4.5	-	-	< 0.001*
Openness	-	-	34.2 ± 2.7	-	-	-	-	34.0 ± 2.9	-	-	0.4

PMDD, Premenstrual dysphoric disorder; BFI, big five personality traits inventory; s.d., standard deviation.

* , Statistically significant.

Regarding the menstrual history of the studied students, PMDD was associated with the early age of menarche (*p*-value < 0.001). In addition, PMDD was associated with an average length of one cycle less than 28 days (*p*-value < 0.001), high MBL, presence of dysmenorrhea (*p*-value < 0.001) and positive family history of PMS (*p*-value 0.003).

Premenstrual dysphoric disorder was significantly associated with BFI personality traits as PMDD was related to low extroversion and agreeableness scores. On the other hand, PMDD was associated with a high neuroticism score.

Binary logistic regression model revealed that significantly related PMDD risk factors include being a medical student, having a duration of menstrual bleeding ≥ 7 days, the average length of one cycle ˂ 28 days, high MBL, presence of dysmenorrhea and positive family history of PMS (sister/mother). Regarding personality traits, low extroversion and agreeableness, and high neuroticism were also significant PMDD risk factors (Table 4).

**TABLE 4:** Binary logistic regression analysis of independent variables significantly associated with the premenstrual dysphoric disorder.

Variables	B	s.e.	Wald	Sig.	Odds ratio	95% CI
Age	0.69	0.74	0.89	0.3	2.0	0.47–8.5
Medical student	1.9	0.84	5.2	0.02*	5.6	1.1–10.8
Urban resident	1.2	0.77	2.5	0.1	3.4	0.75–15.5
Socio-economic class	0.66	0.73	0.83	0.3	1.9	0.46–8.1
Body mass index ≥ 30 Kg/m^2^	1.1	0.67	2.7	0.1	4.1	0.78–21.4
Age of menarche	0.74	0.92	0.65	0.7	1.0	0.94–11.1
Duration of menstrual bleeding ≥ 7 days	1.79	0.78	5.3	0.02*	5.9	1.4–17.6
Average length of one cycle ˂ 28 days	1.38	0.59	5.5	0.01*	6.3	1.3–36.4
High Menstrual bleed loss (MBL-score ˃ 4)	1.7	0.61	8.6	0.003*	7.9	1.8–24.1
Presence of dysmenorrhea	1.8	0.83	4.9	0.02*	6.0	1.2–31.8
Positive family history of premenstrual syndrome (sister/mother)	1.6	0.64	6.2	0.01*	6.6	1.9–38.5
Extroversion	−1.97	0.57	11.9	< 0.001*	0.16	0.02–0.74
Agreeableness	−2.7	0.61	19.6	< 0.001*	0.18	0.07–0.87
Neuroticism	3.78	0.80	22.3	< 0.001*	20.3	6.8–41.9

s.e., standard error; Sig., significant; CI, confidence interval.

* , Statistically significant.

## Discussion

This study found that the prevalence of PMDD among female university students was 21.1%. The results infer that the prevalence rate of PMDD in this study is high compared with 13.4% observed in another previous Egyptian study,^11^ and higher than that in other studies sharing almost the same cultural backgrounds, such as in Kuwait (5.6%),^20^ Jordon (7.7%)^12^ and UAE (16.4%).^21^ Other available results from Pakistan had analogous PMDD prevalence (18.2% – 27.3%).^22,23^ In India, the prevalence of PMDD ranged from 8.0% to 11.1%.^24,25,26,27,28^ The American Psychological Association (APA) reported prevalence of PMDD between 1.8% and 5.8%.^29^ Other studies conducted in the United States and Europe have also reported a much lower prevalence of PMDD.^30,31,32,33^ Korean and Chinese studies have also reported a lower prevalence (2.4% and 2.1% respectively)^34,35^ In Iran, prevalence of PMDD ranged from 6.1% to 15.4%.^8,36,37^ On the other hand, studies from African countries reported higher rates of PMDD. In Ethiopia, prevalence of PMDD ranged from 27.0% to 66.9%.^38,39,40,41^ The reported prevalence from a Nigerian study for PMDD was 38.3%.^42^ A Moroccan study reported the prevalence of PMDD was 50.2%.^43^

The PMDD rates’ inconsistency is probably related to the sample size and characteristics in this study compared with the targeted samples from other studies’. Another possible explanation for this difference could be because of limitations and differences in the definition of PMDD and the usage of different data-collection tools. The American College of Obstetricians and Gynaecologists acknowledges that one of the physical symptoms and mood/behavioral symptoms must be present to diagnose PMDD.^44^ But according to DSM-5, diagnosis can be made even in the absence of physical symptoms if the other symptoms are present in significant severity.^29^

In this study, the most frequently reported premenstrual symptoms were overeating/food cravings (84.2%), fatigue/lack of energy (83.6%), depressed mood/hopelessness (82.0%) and hypersomnia (78.9%). These findings are also approximately concordant with previous findings. Tenkir et al.,^39^ Tschudin et al.,^32^ Kamat et al.^28^ and Seedhom et al.,^11^ reported that more than 80% of university girls had at least one symptom with any degree of severity. Seedhom et al.^11^ stated that severe premenstrual symptoms were fatigue and psychological symptoms (mood swings, anxiety and irritability), while appetite change was not common. According to Hashim et al.,^45^ the most frequently reported premenstrual symptoms were depressed mood, lethargy/fatigue/decreased energy, muscle, joint, abdominal, and back pain, feelings of anger and craving for certain foods.

In the current study, physical symptoms are not the most frequent, but they are severe in 19.6% of the study participants. These findings were consistent with the works of Amiri et al.^37^ and Mchichi et al.^43^ Moreover, Qiao et al.^35^ reported that the most common symptoms were irritability, breast tenderness, depression, abdominal bloating and angry outbursts (59.62%). According to Pearlstein et al.^46^ and Nisar et al.,^47^ the order of frequency of symptoms was anger, irritability, anxiety, tiredness, difficulty in concentration, mood swings and physical symptoms. Some studies reported that physical symptoms are more frequent than psychological symptoms. Pal et al.^23^ and Bansal et al.^26^ found that physical symptoms predominate, and the two most prevalent clinical symptoms noted were muscle pain and lack of energy. Breast pain/tenderness was not common. Ikeako et al.^42^ reported that pelvic discomfort and breast fullness were the most common physical symptoms, while mood changes did not come first.

In this study, the premenstrual symptoms interfered mainly with family relationships followed by relationships with colleagues, social life activities and studying efficiency or productivity (severe in 18.7%, 17.1%, 17.1% and 16.0%, respectively). Home responsibilities were the least to be affected (severe in 3.0% of the study participants). These findings were consistent with Sadler et al.,^48^ Kamat et al.,^28^ Ikeako et al.,^42^ and Bansal et al.,^26^ as they reported that PMDD adversely affects the relationship with family members, social functions, and educational performance. The findings confirm previous findings that negative mood states (dysphoria) have a significant adverse association among university students with academic performance. The academic performance of students is also more likely to deteriorate if they suffer from PMDD. Oral et al.,^49^ Tenkir et al.,^39^ Neumann et al.,^50^ Lyubomirsky et al.,^51^ Omu et al.,^20^ Tolossa et al.^40^ and Shehadeh et al.,^12^ found that PMDD interferes with academic achievement, studying efficiency or productivity.

The regression model revealed that significantly related PMDD risk factors include being a medical student, having a duration of menstrual bleeding ≥ 7 days, the average length of one cycle ˂ 28 days, high MBL, presence of dysmenorrhea and a positive family history of PMS (sister/mother).

Dysmenorrhea and a family history of PMS were significantly associated factors in most related studies as in the studies by Rizk et al.,^21^ Sadler et al.,^48^ and Tsegaye & Getachew.^38^ Other menstrual factors, for example lower age at menarche, the regularity of menstruation, the average length of one cycle of menstruation, and menorrhagia are also reported in previous studies like Balaha et al.,^52^ Kamat et al.^28^ Tolossa et al.,^40^ and Shiferaw & Mamo.^41^

Medical students had significantly more PMDD than commerce college students. This finding is consistent with those of Shehadeh et al.^12^ The reason could be the stressful life of the medical students. Types and levels of stressors, the cultural perspective of girls’ roles and obligations at college age and sociocultural factors that play a crucial role in the production of PMDD are expected to lead to differences in PMDD rates. According to Cohen et al.,^53^ university students lack awareness of PMDD and PMS because their familiarity with menstrual problems is considered immature; being young often leads to mixed psychobiological signs and symptoms of this question. Besides, because female university students are continuously aiming for higher academic achievements, they view academic life as a stressful situation that causes more psychological disturbances. Therefore, the academic-related stressors among university students are another potential correlate for high PMDD and PMS rates.

Some studies reported that obesity, physical inactivity, and nutritional habits were significant risk factors like Kamat et al.,^28^ Hong et al.,^34^ and Seedhom et al.^11^

The current study showed that PMDD was associated with a low extroversion score in terms of personality traits. This result is in line with the findings of the studies conducted by Hallman et al.^54^ In our results, PMDD was associated with high neuroticism score. Neuroticism is the trait-like tendency to experience frequent negative emotions and a perceived inability to cope in response to stress.^55^ High neuroticism in PMDD was reported by Hallman et al.,^55^ Hunter,^56^ Eissa,^57^ Siahbazi et al.,^58^ Singh et al.,^24^ Arslantaş et al.,^59^ Izadi & Amiri.^8^ We also found that students suffering from PMDD were associated with lower scores in agreeableness. Agreeableness reflects the individual differences in cooperation and social harmony. The association between agreeableness and PMDD may be influenced by social support; more significant social support enjoyed by women with a higher degree of agreeableness may be associated with less risk of developing PMDD or decreased incidence of premenstrual problems over time.^54^ Low agreeableness in PMDD was reported by Hunter^56^ and Izadi & Amiri.^8^

There was no association between conscientiousness and PMDD in the current study, while in the work of Arslantaş et al.,^59^ PMDD was associated with a low conscientiousness score. Our results also did not show a significant association between the openness dimension and PMDD. By contrast, Freeman et al.^60^ reported higher scores on the novelty-seeking dimension (which has analogy with openness) in women with severe PMS compared to a normative female sample.

## Strengths and limitations

This cross-sectional study permitted relationship, but not causality, to be studied.

## Conclusion

Based on the results of the current study, PMDD was highly prevalent among university students. Medical students, prolonged menstrual bleeding, the average length of one cycle ˂28 days, menorrhagia, dysmenorrhea, and positive family history of the PMS were factors that were significantly correlated with PMDD. Regarding personality traits, low extroversion and agreeableness and high neuroticism were also significant PMDD risk factors.

Implementing a reproductive health programme into the school and college health education programme was recommended to help provide students with reliable and up-to-date information, education and support on reproduction in general and menstrual problems in particular.

Girls must receive care. Many girls may feel shy and may be unable to disclose that they suffer from PMS and, as a result, do not seek medical advice and this can have a detrimental effect on both academic life and educational accomplishments, quality of life and daily living activities. Asking regarding PMS and screening for PMS to provide treatment if appropriate is one of the functions of healthcare providers in the related institutions.

## References

[1] Biggs WS, Demuth RH. Premenstrual syndrome and premenstrual dysphoric disorder. Am Fam Phys [serial online]. 2011 [cited 2020 Sept 1] 84(8):918–924. Available from: www.aafp.org/afp 22010771

[2] Roehr B. American psychiatric association explains DSM-5. BMJ. 2013 6 6;346:f3591. 10.1136/bmj.f3591 23744600

[3] Le J, Thomas N, Gurvich C. Cognition, the menstrual cycle, and premenstrual disorders: A review. Brain Sci. 2020 4;10(4):198. 10.3390/brainsci10040198 PMC7226433 32230889

[4] Wakefield JC. DSM-5: An overview of changes and controversies. Clin Soc Work J. 2013 6 1;41(2):139–154. 10.1007/s10615-013-0445-2

[5] Anson O. Exploring the bio-psycho–social approach to premenstrual experiences. Soc Sci Med. 1999 7 1;49(1):67–80. 10.1016/S0277-9536(99)00079-9 10414841

[6] Farage MA, Osborn TW, MacLean AB. Cognitive, sensory, and emotional changes associated with the menstrual cycle: A review. Arch Gynecol Obstet. 2008 10;278(4):299–307. 10.1007/s00404-008-0708-2 18592262

[7] Izadi M, Amiri S. Personality characteristics in female students with premenstrual dysphoric disorder and premenstrual syndrome. Adv Nurs Midwifery. 2019;28(3):40–45. 10.29252/anm-280307

[8] Ciarrochi J, Deane FP, Anderson S. Emotional intelligence moderates the relationship between stress and mental health. Personal Individ Diff. 2002 1 19;32(2):197–209. 10.1016/S0191-8869(01)00012-5

[9] Storrie K, Ahern K, Tuckett A. A systematic review: Students with mental health problems – A growing problem. Int J Nurs Pract. 2010 2;16(1):1–6. 10.1111/j.1440-172X.2009.01813.x 20158541

[10] Seedhom AE, Mohammed ES, Mahfouz EM. Life style factors associated with premenstrual syndrome among El-Minia University Students, Egypt. Int Sch Res Notices. 2013;2013:617123. 10.1155/2013/617123

[11] Shehadeh JH, Hamdan-Mansour AM. Prevalence and association of premenstrual syndrome and premenstrual dysphoric disorder with academic performance among female university students. Perspect Psychiatr Care. 2018 4 1;54(2):176–184. 10.1111/ppc.12219 28543046

[12] Center for Disease Control and Prevention. Epi InfoTM | CDC [homepage on the Internet]. 2013 [cited 2019 Jun 12]. Available from: https://www.cdc.gov/epiinfo/index.html

[13] Google privacy and terms [homepage on the Internet]. [cited 2021 Jan 12] Available from: https://policies.google.com/privacy?hl=en

[14] El-Gilany A, El-Wehady A, El-Wasify M. Updating and validation of the socio-economic status scale for health research in Egypt. East Mediterr Health J. 2012 9 1;18(9):962–968. 10.26719/2012.18.9.962 23057390

[15] Toxqui L, Pérez-Granados AM, Blanco-Rojo R, Wright I, Vaquero MP. A simple and feasible questionnaire to estimate menstrual blood loss: Relationship with hematological and gynecological parameters in young women. BMC Womens Health [serial online]. 2014 [cited 2019 Apr 27];14(1):1–6. Available from: http://www.biomedcentral.com/1472-6874/14/71 10.1186/1472-6874-14-71 PMC4046034 24886470

[16] Rosenberg DE, Norman GJ, Wagner N, Patrick K, Calfas KJ, Sallis JF. Reliability and validity of the Sedentary Behavior Questionnaire (SBQ) for adults. J Phys Act Health. 2010 11 1;7(6):697–705. 10.1123/jpah.7.6.697 21088299

[17] Özdel K, Kervancıoğlu A, Taymur İ, et al. Premenstrual Symptom Screening Tool: A useful tool for DSM-5 premenstrual dysphoric disorder. J Clin Anal Med. 2015;6:581–585. 10.4328/JCAM.2314

[18] John OP, Srivastava S. The big-five trait taxonomy: History, measurement, and theoretical perspectives. Berkeley: University of California; 1999.

[19] IBM SPSS Statistics for Windows, Version 27 [homepage on the Internet]. Armonk, NY: IBM Corp.; 2020 [cited 2020 Oct 4]. Available from: http://www-01.ibm.com/support/docview.wss?uid=swg27049428

[20] Omu FE, Al-Marzouk R, Delles H, Oranye NO, Omu AE. Premenstrual dysphoric disorder: Prevalence and effects on nursing students’ academic performance and clinical training in Kuwait. J Clin Nurs. 2011 10;20(19–20):2915–2923. 10.1111/j.1365-2702.2011.03708.x 21362077

[21] Rizk DE, Mosallam M, Alyan S, Nagelkerke N. Prevalence and impact of premenstrual syndrome in adolescent schoolgirls in the United Arab Emirates. Acta Obstet Gynecol Scand. 2006 5;85(5):589–598. 10.1080/00016340600556049 16752239

[22] Tabassum S, Afridi B, Aman Z, Tabassum W, Durrani R. Premenstrual syndrome: Frequency and severity in young college girls. Anxiety. 2005 12;45(27.3):4–5. 16438276

[23] Pal SA, Dennerstein L, Lehert P. Premenstrual symptoms in Pakistani women and their effect on activities of daily life. JPMA J Pak Med Assoc. 2011 8 1;61(8):763. 22355998

[24] Singh C, Jain J, Singh K, Jain M, Chaudhary A. A study of premenstrual dysphoric disorder prevalence, phenomenology and personality factors in college going students. Indian J Health Wellbeing. 2016 10 1;7(10):962. 10.4103/0019-5545.183796

[25] Koganti CT, Bobba NS. A study on the prevalence of premenstrual dysphoric disorder in medical students. Acad J Med. 2020 1 3(1):74–77. 10.47008/ajm.2020.3.1.15

[26] Bansal D, Raman R, Rao TS. Premenstrual dysphoric disorder: Ranking the symptoms and severity in Indian college students. J Psychosexual Health. 2019 4;1(2):159–163. https://doi.org/10.1177%2F2631831819827183

[27] Banerjee N, Roy KK, Takkar D. Premenstrual dysphoric disorder – A study from India. Int J Fertil Womens Med. 2000 9 1;45(5):342–344. 11092706

[28] Kamat SV, Nimbalkar AS, Nimbalkar SM. 465 premenstrual syndrome in adolescents of Anand-cross-sectional study from India using premenstrual symptoms screening tool for adolescents (Psst-A). Arch Dis Childhood [serial online]. 2012 [cited 2020 Dec 29];97(Suppl. 2):A136. Available from: http://adc.bmj.com/

[29] American Psychiatric Association. Diagnostic and statistical manual of mental disorders (DSM-5^®^). Washington: American Psychiatric Pub; 2013.

[30] Soares CN, Cohen LS, Otto MW, Harlow BL. Characteristics of women with premenstrual dysphoric disorder (PMDD) who did or did not report history of depression: A preliminary report from the Harvard Study of Moods and Cycles. J Womens Health Gender-based Med. 2001 11 1;10(9):873–878. 10.1089/152460901753285778 11747682

[31] Wittchen HU, Becker E, Lieb R, Krause P. Prevalence, incidence and stability of premenstrual dysphoric disorder in the community. Psychol Med. 2002;32(1):119. 10.1017/S0033291701004925 11883723

[32] Tschudin S, Bertea PC, Zemp E. Prevalence and predictors of premenstrual syndrome and premenstrual dysphoric disorder in a population-based sample. Arch Womens Mental Health. 2010 12;13(6):485–494. 10.1007/s00737-010-0165-3 20449618

[33] Rojnić Kuzman M, Hotujac L. Premenstrual dysphoric disorder – A neglected diagnosis? Preliminary study on a sample of Croatian students. Coll Antropol. 2007 1 4;31(1):131–137. 17598391

[34] Hong JP, Park S, Wang HR, et al. Prevalence, correlates, comorbidities, and suicidal tendencies of premenstrual dysphoric disorder in a nationwide sample of Korean women. Soc Psychiatry Psychiatr Epidemiol. 2012;47(12):1937–1945. 10.1007/s00127-012-0509-6 22538387

[35] Qiao M, Zhang H, Liu H, et al. Prevalence of premenstrual syndrome and premenstrual dysphoric disorder in a population-based sample in China. Eur J Obstet Gynecol Reprod Biol. 2012 5 1;162(1):83–86. 10.1016/j.ejogrb.2012.01.017 22377229

[36] Zareipour MA, Movahed E, Jadgal KM, Jabari B, Amery M. Evaluation of the frequency of clinical manifestations of premenstrual syndrome in young married women in Kerman. Curr Res Med Sci. 2017;2(1):11–18.

[37] Amiri Farahani L, Farokhi F, Abbasi A. Prevalence, severity, and clinical manifestations of premenstrual syndrome among the students residing in the dormitories of Arak University of Medical Sciences, Iran. Qom Univ Med Sci J. 2014 1 10;7(6):34–40.

[38] Tsegaye D, Getachew Y. Premenstrual dysphoric disorder and associated factors among female health science students in Wollo University, Ethiopia, 2017/18. Matern Health Neonatol Perinatol. 2019 12;5(1):1–8. 10.1186/s40748-019-0102-z 31139430 PMC6530005

[39] Tenkir A, Fisseha N, Ayele B. Premenstrual syndrome: Prevalence and effect on academic and social performances of students in Jimma University, Ethiopia. Ethiop J Health Dev. 2003;17(3):181–188.

[40] Tolossa FW, Bekele ML. Prevalence, impacts and medical managements of premenstrual syndrome among female students: Cross-sectional study in college of health sciences, Mekelle University, Mekelle, Northern Ethiopia. BMC Womens Health [serial online]. 2014 [cited 2020 Dec 29];14(1):1–9. Available from: http://www.biomedcentral.com/1472-6874/14/52 10.1186/1472-6874-14-52 PMC3994244 24678964

[41] Shiferaw MT, Mamo Wubshet DT. Menstrual problems and associated factors among students of Bahir Dar University, Amhara National Regional State, Ethiopia: A cross-sectional survey. Pan Afr Med J. 2014;17:246. https://doi.org/10.11604%2Fpamj.2014.17.246.2230 25309646 10.11604/pamj.2014.17.246.2230 PMC4189866

[42] Ikeako LC, Ezegwui HU, Nwafor MI, Nwaogu-Ikeojo EE. Pattern of premenstrual symptoms among pre-clinical medical students at the University of Nigeria. Orient J Med. 2014 6 6;26(1–2):52–57.

[43] Mchichi Alami K, Tahiri S, Moussaoui D, Kadri N. Évaluation des symptômes dysphoriques prémenstruels dans une population de femmes à Casablanca. L’Encéphale (Paris) [serial online]. 2002 [cited 2020 Dec 29];28(6):525–530. Available from: http://pascal-francis.inist.fr/vibad/index.php?action=getRecordDetail&idt=14434704 12506265

[44] Henderson CW. ACOG issues guidelines on diagnosis and treatment of PMS. Womens Health Wkly. 2000;5(6):20–21.

[45] Hashim MS, Obaideen AA, Jahrami HA, et al. Premenstrual syndrome is associated with dietary and lifestyle behaviors among university students: A cross-sectional study from Sharjah, UAE. Nutrients. 2019 8;11(8):1939. 10.3390/nu11081939 PMC6723319 31426498

[46] Pearlstein T, Yonkers KA, Fayyad R, Gillespie JA. Pretreatment pattern of symptom expression in premenstrual dysphoric disorder. J Affect Disord. 2005 4 1;85(3):275–282. 10.1016/j.jad.2004.10.004 15780697

[47] Nisar N, Zehra N, Haider G, Munir AA, Sohoo NA. Frequency, intensity and impact of premenstrual syndrome in medical students. J Coll Physicians Surg Pak. 2008 8 1;18(8):481. 18798584

[48] Sadler C, Smith H, Hammond J, et al. Lifestyle factors, hormonal contraception, and premenstrual symptoms: The United Kingdom Southampton Women’s Survey. J Womens Health. 2010 3 1;19(3):391–396. 10.1089/jwh.2008.1210 PMC3091016 20156129

[49] Oral E, Kirkan T, Yazici E, Cansever MG, Aydin N. Premenstrual symptom severity, dysmenorrhea, and school performance in medical students. Psychiatry Behav Sci. 2012 10 1;2(4):143. 10.5455/jmood.20120912035016

[50] Neumann G, Olitsky N, Robbins S. Job congruence, academic achievement, and earnings. Labour Econ. 2009 10 1;16(5):503–509. 10.1016/j.labeco.2009.03.004

[51] Lyubomirsky S, Kasri F, Zehm K. Dysphoric rumination impairs concentration on academic tasks. Cogn Ther Res. 2003 6;27(3):309–330.

[52] Balaha M, Amr MA, Moghannum M, Muhaida N. The phenomenology of premenstrual syndrome in female medical students: A cross sectional study. Pan Afr Med J. 2010;5(1):4. 10.4314/pamj.v5i1.56194 21120003 PMC2984319

[53] Cohen LS, Soares CN, Otto MW, Sweeney BH, Liberman RF, Harlow BL. Prevalence and predictors of premenstrual dysphoric disorder (PMDD) in older premenopausal women: The Harvard study of moods and cycles. J Affect Disord. 2002 7 1;70(2):125–132. 10.1016/S0165-0327(01)00458-X 12117624

[54] Hallman J, Oreland L, Edman G, Schalling D. Thrombocyte monoamine oxidase activity and personality traits in women with severe premenstrual syndrome. Acta Psychiatr Scand. 1987 9;76(3):225–234. 10.1111/j.1600-0447.1987.tb02890.x 3673649

[55] Barlow DH, Sauer-Zavala S, Carl JR, Bullis JR, Ellard KK. The nature, diagnosis, and treatment of neuroticism: Back to the future. Clin Psychol Sci. 2014 5;2(3):344–365. https://doi.org/10.1177%2F2167702613505532

[56] Hunter MS. A biopsychosocial approach to premenstrual problems. In: Cockburn J, Pawson ME, editors. Psychological challenges in obstetrics and gynecology. London: Springer, 2007; 255–262.

[57] Eissa M. Personality and psychosocial factors affecting premenstrual syndrome. Curr Psychiatry. 2010 1;17(1):55–62.

[58] Siahbazi SH, Hariri F, Montazeri A, Moghadam Banaem L. Standardization of premenstrual symptoms screening questionnaire PSST: Translation and psychometric Iranian species. J Payesh. 2011;10(4):421–427.

[59] Arslantaş H, Abacigil F, Çinakli Ş. Relationship between premenstrual syndrome and basic personality traits: A cross-sectional study. Sao Paulo Med J. 2018 8;136(4):339–345. 10.1590/1516-3180.2018.0061240418 30110077 PMC9881699

[60] Freeman EW, Schweizer E, Rickels K. Personality factors in women with premenstrual syndrome. Psychosom Med. 1995 9 1;57(5):453–459. 10.1097/00006842-199509000-00007 8552736

